# 

**Published:** 1900-02

**Authors:** 


### CONTENTS.

ORIGINAL COMMUNICATIONS—

RELATIVE DIGESTIBILITY OF BREADS MADE WITH DIFFERENT LEAVENING AGENTS. By Edgar Everhart, Ph.D., Atlanta, Ga. .......... 793
SOME CASES IN CONSERVATIVE GYNECOLOGY. By G. Betton Massey, M.D., Philadelphia, Pa. .......... 802
MEMBRANOUS OBSTRUCTION OF THE LARYNX. By W. Jay Bell, M.D., Atlanta, Ga. .......... 807
WHOOPING-COUGH OF INFANTS, AND TREATMENT TO PREVENT COMPLICATIONS. By C. E. Murphey, M.D., Atlanta, Ga. .......... 811

SOCIETY REPORTS—

ATLANTA SOCIETY OF MEDICINE .......... 816

CORRESPONDENCE—

LETTER FROM DR. RIDLEY .......... 824

EDITORIAL NOTES AND COMMENTS—

THE RECENT VETO OF THE OSTEOPATHY BILL .......... 828

QUACKERY IN GEORGIA .......... 831

THE ANTI-VIVISECTION MOVEMENT .......... 834

THE HUMAN ODOR .......... 835

"WORDS, WORDS, WORDS" .......... 836

NEWS AND NOTES .......... 839
CONTENTS CONTINUED .......... x

